# Morbidity associated with treatment of chronic anal fissure

**DOI:** 10.12669/pjms.295.3623

**Published:** 2013

**Authors:** Ansar Latif, Anila Ansar, Muhammad Qasim Butt

**Affiliations:** 1 Dr. Ansar Latif, MBBS, FCPS, Assistant Professor, Department of Surgery, Islam Medical College, Sialkot, Pakistan.; 2 Dr. Anila Ansar, MBBS, MCPS, FCPS, Assistant Professor, Department of Obstetrics and Gynaecology, Islam Medical College, Sialkot, Pakistan.; 3 Dr. Muhammad Qasim Butt, MBBS, FCPS, Department of Surgery, Combined Military Hospital, Kohat, Pakistan.

**Keywords:** Chemical sphincterotomy, Internal sphincterotomy, Pain relief, Topical glyceryl trinitrate

## Abstract

***Objective: ***To assess the effectiveness of different modes of treatment of chronic anal fissure as regards improvement of symptoms and complications.

***Methods: ***This prospective study included 129 consecutive patients with chronic anal fissures presented to the Surgical Outpatients’ Department of Islam Teaching Hospital Sialkot, Pakistan; from September 2010 to November 2012. Patients were distributed in three groups. In “OBG group”, patients had attended Gynae/Obs OPD and got treated and were then referred to surgical OPD for failure of treatment or recurrence. Patients who presented with history of treatment by GPs were included in “GP Group” “SGR Group” included those who directly reported to surgical OPD for treatment. Patients were managed both pharmacologically as OPD patients and surgically as admitted patients. Patients were instructed to apply small amounts of 0.2% GTN paste in soft white paraffin, to the anoderm with finger tips three times a day. Patients were evaluated at two-week intervals and at each visit the symptoms control, adverse effects and fissure status were recorded. If there was symptomatic relief or the fissure healing was in progress, the treatment was continued for a total duration of eight weeks. Operated patients were nursed in wards after surgery i.e Internal Anal Sphicterotomy. They were advised to report to OPD weekly for one month or earlier if they experienced any symptoms suggestive of complications. Patients were declared cured in case of complete symptomatic relief with fissure healing. Success, failure and associated problems were recorded and analysed to get results.

***Results:***This study included 129 patients who could be followed up for a minimum of three months. These patients were referred by gynaecologist i.e. 22 (17%) for treatment failure while 5 patients with wrong diagnosis were not included in statistical analysis; similarly 41 (32%) patients were referred by general practitioners and 9 patients with wrong diagnosis were excluded. Sixty six patients i.e. 51% were those who directly reported to surgical OPD and had no previous treatment. With surgical treatment, pain, bleeding per rectum and constipation showed significant improvement as compared to GTN ointment application. Fissure healing was 100% in surgical group as compared to 74% in medical group. Complications were recorded and were found to be headache with medical treatment; while the most feared complication with surgical treatment i.e. permanent incontinence was not encountered in our study.

***Conclusion: ***Topical glyceryl trinitrate is economical, has a good healing rate, and faecal incontinence has not been reported. Its effectiveness, however, depends on patients’ compliance which may be poor in view of associated headaches and a local burning sensation. It is first line of treatment for anal fissure but lateral internal sphincterotomy is superior, more effective and curative than the chemical sphincterotomy. Surgery is reserved for people with anal fissure who have tried medical therapy for at least one to three months but failed.

## INTRODUCTION

Anal fissure is a painful linear wound in the squamous epithelium of the anal canal distal to the dentate line. The incidence of anal fissures is around 1 in 350 adults. They occur equally in men and women and most often occur in young adults aged 15 to 40. It is usually located in the posterior midline but occurs anteriorly in a fifth or more or patients. It typically causes pain during defecation which may last for 1–2 hour afterwards.^1^ The finding on physical examination is spasm of the anal canal due to hypertonia of the internal anal sphincter. It has been postulated that this may either be due to or be the result of ischaemia.^2^

Most anal fissures are caused by stretching of the anal mucosa beyond its capability. In adults, fissures may be caused by constipation, the passing of large, hard stools, or by prolonged diarrhea as well as anal sex. Other causes of anal fissures include: childbirth trauma in women, Crohn'sHYPERLINK "http://en.wikipedia.org/wiki/Crohn%27s_disease" disease and poor toileting in young children.^3^

 Fissures are defined as acute if present for less than 6 weeks, and they are defined as chronic if present for more than 6 weeks.^4^ Anal fissures may present with rectal pain described as burning, cutting, or tearing that occurs during defecation. Bright-red blood appears on the surface of stools and is present only in a small amount. Occasionally, blood is found on toilet paper after wiping. The patient may report no bleeding.^5^

The management aims to reduce anal tone. The initial approach is non-operative. Acute fissures rarely require surgical intervention and usually improve with conservative management. Symptoms from an acute fissure often resolve within 10-14 days of conservative medical management; however, as long as 6-8 weeks may be necessary for the actual tear to heal.^6^ After 6-8 weeks, the fissure is considered chronic, and more active measures such as chemical or surgical sphincterotomy may be considered. Among conservative treatment, diltiazem, nifedipin, and nitroglycerine can be considered as first-line treatments. Topical application of glyceryl trinitrate (GTN) ointment to the anal rim temporarily reduces resting anal sphincter pressure and promotes healing of fissures whilst minimising the risk of incontinence; however nitroglycerine has a significant risk of headaches.^7^ Isosorbide dinitrate is an alternative nitric oxide donor that has been used successfully in the treatment of anal fissures. Botulinum toxin, a neurotoxin released from Clostridium botulinum (BTX injections) is also used to treat refractory cases.^8^

Surgical therapy is often reserved when conservative treatment fails to heal anal fissures. Surgery includes lateral sphincterotomy, advancement flap procedures and fissurectomy. By 1959, the ‘standard internal sphincterotomy’ comprised division of only half of the IAS (internal anal sphincter) to the dentate line in its lateral or posterolateral part. Lateral sphincterotomy diminishes internal anal sphincter tone and thereby reduces anal canal pressure. This improves anal mucosal blood flow and promotes the healing of anal fissures. Apart from the associated risks and discomfort, surgery can cause incontinence.^9^

Anal advancement flap is indicated for patients with primary or recurrent fissures and for women with a complicated obstetric history with low resting anal canal pressure. This operation avoids further disruption to the internal sphincter and avoids factors that might otherwise jeopardise continence.^10^

Manual dilatation of the anus is a simple procedure and previously a popular treatment. Incontinence is a concern and current opinion is that manual dilatation of the anus for the treatment of anal fissures is not recommended.^11^

 Fissures associated with pregnancy are commonly located anteriorly and are often associated with low anal canal pressures. These secondary fissures are most appropriately treated by addressing the underlying disease process.^12^

Patients with anal fissures are in regular attendance in surgical OPD; most of which have already had treatment from and/ or referred by general physicians or gynaecologists/ obstetricians; for treatment failure, inadequate treatment or wrong treatment because of misdiagnosis. This study was planned to see the factors involved and success of treatment carried by the surgeons in Islam Teaching Hospital, Sialkot, Pakistan.

## METHODS

This prospective study included 129 consecutive patients with acute and chronic anal fissures presented to the Surgical Outpatients’ Department of Islam Teaching Hospital Sialkot, Pakistan; from September 2010 to November 2012. These patients had persistent, symptomatic anal fissures. Informed consent was obtained from all the patients after an explanation of the nature of the disease, treatment method and the possible unwanted effects. Patients were distributed in three groups. In “OBG group”, patients had attended Gynae/Obs OPD and got treated during and after pregnancy and were then referred to surgical OPD for failure of treatment or recurrence. Patients who presented with history of treatment by the General practitioners were included in “GP Group” who had failure of pharmacological treatment or recurrence and were referred to surgical OPD for treatment. The patients were grouped as “SGR Group” who directly reported to surgical OPD for treatment.

Patients were managed both pharmacologically as OPD patients and surgically as admitted patients. They were instructed to apply small amounts of 0.2% GTN paste in soft white paraffin, to the anoderm with finger tips three times per day. All patients were encouraged to take high fibre diet, warm sitz baths twice a day and warned about the possibility of headache and dizziness. Patients were evaluated at two-week intervals and at each visit the symptoms’ control, adverse effects and fissure status were recorded. If there was symptomatic relief or the fissure healing was in progress, the treatment was continued for a total duration of eight weeks. Afterwards, the patients were given the option to resume the treatment in case of recurrence or abandon this therapy and consider surgical intervention. Two follow up visits, at two month interval, were arranged after the completion of the initial therapy to establish the long-term effectiveness of 0.2% GTN ointment.

 Operated patients were nursed in wards thereafter. Postoperative analgesia with Tablet Tramadol 50 mg. Analgesic tablets were dispensed strictly on demand by patient. The patient was allowed full oral feed six hours after surgery. They were advised Sitz bath three times daily and stool softener (Syrup Cremaffin, 2 tsp thrice daily).They were advised to report to OPD routinely at weekly intervals for a period of one month or earlier if they experienced any symptoms suggestive of complications. Patients were declared cured in case of complete symptomatic relief with fissure healing.

Patients with history of previous surgery in the anal canal, inflammatory bowel disease, HIV infection and those with cardiac disease using oral or sublingual nitrates were excluded from this study. The SPSS 17 software was used for data analysis.

## RESULTS

In this study, 129 patients were included who could be followed up for a minimum of three months after completing their treatment. These patients were referred by gynaecologist i.e. 22 (17%) for treatment failure while 5 patients with wrong diagnosis were not included in statistical analysis; similarly 41 (32%) patients were referred by general practitioners and 9 patients with wrong diagnosis were excluded from statistical analysis. Sixty six patients i.e. 51% were those who directly reported to surgical OPD and had no previous treatment. With surgical treatment, pain, bleeding per rectum and constipation showed significant improvement as compared to GTN ointment application. Fissure healing was 100% in surgical group as compared to 74% in medical group. Collective data is shown in Table-I.

**Table-I:** General demographic data all cases

Total Patients	129
OBG group	22(17%)
GP group	41(32%)
SGR group	66(51%)
Age	20-49 ( mean 30 years)
Sex (M:F)	83:46
History of pain	129(100%)
History of bleeding per rectum	93(72%)
History of constipation	85(66%)
Passing hard stools	80(62%)
Medical treatment(0.2% GTN ointment)	129 (100%)
Surgical treatment	33(25.58%)

Out of twenty two patients referred by obstetricians, 18 (82%) were treated by chemical method with success and only revision of the treatment taken previously and counselling of proper application could lead to cure and only 4 (18%) patients were shifted to surgical management. In GP group patients 28 (68%) out of forty one patients were successfully treated by 0.2% GTN ointment while 13 (32%) patients had to undergo lateral internal sphincterotomy. Surgical OPD group patients were mostly treated by chemical method i.e. 50 (76%) and 16(24%) patients were treated surgically. Complications and morbidity encountered is shown in Table-II.

**Table-II:** Statistics of morbidity in both groups

Medical treatment (0.2% GTN ointment) Patients=129	Pain relief	90	70%
	Headache	129	100%
	Patient requiring treatment of headache	77	60%
	Success rate	96	74.4%
	Failure/recurrence	33	25.5%
Surgical treatment Patients = 33	Pain relief	33	100%
	Faecal soiling	2	6%
	Flatus incontinence	3	9%
	Permanent Faecal incontinence	0	0
	Bleeding	1	3%
	Haematoma	1	3%
	Itching/burning	3	9%
	Wound infection	1	3%
	Failure/recurrence	0	0

## DISCUSSION

With surgical treatment, pain, bleeding per rectum and constipation showed significant improvement as compared to GTN ointment application. Fissure healing was 100% in surgical group as compared to 74% in medical group. Complications were recorded and were found to be headache with medical treatment; while the most feared complication with surgical treatment i.e. permanent incontinence was not encountered in our study.

The reported efficacy of nitric oxide donors varies widely in the literature (47-88%), depending on the agent used, the duration of treatment, whether the fissure was acute or chronic, and how the success of therapy was measured i.e. symptomatic relief, healed fissure or manometric finding of reduced anal sphincter tone. Lateral internal sphincterotomy has been among the most gratifying surgical interventions for anal fissures but published literature has reported a 2.3% wound infection rate and 0 to 34% incidence of incontinence to flatus and liquid stool following this procedure.^13^

Patients referred by gynaecologists/obstetricians with treatment failure then treated in surgical department highlighted that 5 patients were misdiagnosed and had haemorrhoids and those patients were not included in the statistics; similarly patients who were included in the study had either poor compliance to treatment or their treatment was inadequate which ultimately led their referral to surgical department. Same findings were true for patients referred by general practitioners for which 9 patients were wrongly diagnosed for other perianal pathologies and were treated accordingly but excluded for statistical analysis. Such findings were also highlighted in a study done by Grucela et al^14^ and recommended that improvements be made in physician education concerning common, benign anal disorders.

Out of 129 patients, 96 patients i.e.74.4% patients recovered completely with topical application of 0.2% Glyceryl trinitrate ointment; so the success rate was 74% and failure rate was 25% which is comparable to the study by Khan HU et al^15^ in 2006 which had success rate of 64%. The success and failure rates were different in the study by Memon MR et al^16^ in 2010.

In the study by El Tinay OE et al^17^; Complete symptomatic relief was achieved in all patients within one month of therapy. Two patients, with chronic anal fissures presented with recurrent symptoms within one month of the completion of therapy both of them were successfully treated with repeat glyceryl trinitrate course. Treatment was terminated in six (5.2%) patients: five (4.3%) experienced intolerable adverse effects and one (0.8%) patient failed to respond. All these patients were successfully treated with lateral internal sphincterotomy. In this study, success rate with 0.2% GTN ointment was 74%; while success rate of surgical treatment by internal anal sphicterotomy was 100%.

Two more recent studies from Pakistan have reported that diltiazem hydrochloride is equally effective in healing chronic anal fissure like glyceryl trinitrate. Diltiazem caused fewer side-effects particularly headache than GTN ointment. As such Diltiazem may be the first-line treatment for chemical sphincterotomy for the chronic anal fissure.^18^^,^^19^

Libertiny et al^20^ in a similar comparative trial showed 98% healing of anal fissure with lateral internal sphincterotomy while GTN relieved 56%, with 10% recurrence. Other studies Simpson et al^21^ , Lysy J et al^22^ , Novell F et al^23^, show fissure healing in 66.7% with side effect of headache and a recurrence rate of 25% within six months of topical GTN. Other studies have shown healing rate up to 70% with GTN. Comparison of morbidity with other studies is shown in Table-III.

**Table-III:** Comparison of complications

	*Present study*	*Memon MR et al*	*Lewis et al* ^24^	*Syed et al* ^25^	*Pervinkoff et al* ^26^	*Khubchandani & Reed* ^27^	*Oh* ^28^ * (total patients 1313)*
Success rate with GTN	74%	30%					
Persistence of fissure	25.5%	69%					
Success rate in lateral internal sphincterotomy	100%	100%					18 cases 0f recurrence
Permanent incontinence of faeces	nil	nil			2%		
Incontinence of flatus	6%		17%		8%	35%	21 cases
Faecal soiling	3%					22%	
Itching and burning	9%			2.6%			
Wound infection	3%			nil			

In a study conducted by Siddique MI et al^29^ in Bangladesh; After 8 weeks of complete treatment 25 (80.64%) patients in ointment group were asymptomatic with healed fissure in 21 (67.74%) patients in contrast to surgical sphincterotomy showing 100% pain relief as well as healing of fissure. In both groups symptomatic relief occurred earlier than fissure healing. Two female patients (6%) in surgical group reported to have minor incontinence to flatus on stress but no incontinence was reported in ointment. In this study, success rate with 0.2% GTN ointment was 74%; while success rate of surgical treatment by internal anal sphicterotomy was 100%.

## CONCLUSION

0.2% Glyceryl trinitrate ointment is an effective way of treating acute anal fissure. Chemical sphincterotomy has the advantages of avoiding long term complications (notably incontinence) and not requiring hospitalisation. Topical glyceryl trinitrate is cheap, has a good healing rate, and faecal incontinence has not been reported. Its effectiveness, however, depends on patient compliance which may be poor in view of associated headaches and a local burning sensation. It is first line of treatment for anal fissure but lateral internal sphincterotomy was superior, more effective and curative than the chemical sphincterotomy. Surgery is reserved for people with anal fissure who have tried medical therapy for at least one to three months but failed. It is not the first option in treatment. The main concern with surgery is the development of anal incontinence. But permanent incontinence is rare in expert hands.

## Notes

***Recommendations: ***Improvements should be made in physicians’ education concerning common, benign anal disorders.

***Approval of Ethical committee: ***Approval from ethical committee of Islam teaching hospital affiliated to Islam Medical College was taken for conducting the study.
